# Flagellar Motility During *E. coli* Biofilm Formation Provides a Competitive Disadvantage Which Recedes in the Presence of Co-Colonizers

**DOI:** 10.3389/fcimb.2022.896898

**Published:** 2022-07-08

**Authors:** Wafa Benyoussef, Maxime Deforet, Amaury Monmeyran, Nelly Henry

**Affiliations:** Unité Mixte de Recherche Sorbonne Université, CNRS, Laboratoire Jean Perrin (UMR 8237), Paris, France

**Keywords:** colonization kinetics, biofilm, competition, co-colonization, motility, fluorescence, microscopy, millifluidic channel

## Abstract

In nature, bacteria form biofilms in very diverse environments, involving a range of specific properties and exhibiting competitive advantages for surface colonization. However, the underlying mechanisms are difficult to decipher. In particular, the contribution of cell flagellar motility to biofilm formation remains unclear. Here, we examined the ability of motile and nonmotile *E. coli* cells to form a biofilm in a well-controlled geometry, both in a simple situation involving a single-species biofilm and in the presence of co-colonizers. Using a millifluidic channel, we determined that motile cells have a clear disadvantage in forming a biofilm, exhibiting a long delay as compared to nonmotile cells. By monitoring biofilm development in real time, we observed that the decisive impact of flagellar motility on biofilm formation consists in the alteration of surface access time potentially highly dependent on the geometry of the environment to be colonized. We also report that the difference between motile and nonmotile cells in the ability to form a biofilm diminishes in the presence of co-colonizers, which could be due to motility inhibition through the consumption of key resources by the co-colonizers. We conclude that the impact of flagellar motility on surface colonization closely depends on the environment properties and the population features, suggesting a unifying vision of the role of cell motility in surface colonization and biofilm formation.

## Introduction

Biofilms represent the preferred lifestyle for bacteria (Flemming et al., 2016). In these three-dimensional structures, the aggregated cells prosper in a self-produced polymer extracellular matrix that protects them from shear stress, grazers and biocides (Hall-Stoodley et al., 2004; Karygianni et al., 2020). The formation of a biofilm is a highly multifactorial process in which the cell properties and the details of the environment are both important, bringing about a tremendous diversity in behavior.

In the planktonic state, most bacteria swim in a series of runs and tumbles and rotate their flagella assembled in bundles (Berg and Anderson, 1973; Silverman and Simon, 1974; Nakamura and Minamino, 2019), which provides a significant adaptive advantage in nutrient search (Colin et al., 2021). The importance of this cell motility to biofilm formation has been investigated in a large spectrum of bacterial species and environmental conditions, resulting in differing viewpoints. For example, early research determined that motility was crucial for biofilm development in *Pseudomonas aeruginosa* (O’Toole and Kolter, 1998), *Listeria monocytogenes* (Lemon et al., 2007 *)* and *Escherichia coli* (Pratt and Kolter, 1998; Wood et al., 2006). Furthermore, flagellar motility is often associated with increased virulence in pathogenic species, with motile bacteria exhibiting facilitated host colonization (Josenhans and Suerbaum, 2002).

Different functional mechanisms may actually be involved in motility effect to biofilm formation which do not necessarily engage flagella-assisted adhesion (Haiko and Westerlund-Wikstrom, 2013). Motility *per se* can support aerotaxis, inducing interface accumulation which indirectly promotes biofilm formation (O’Toole and Kolter, 1998; Suchanek et al., 2020). It has also been shown that elongated motile bacteria could accumulate near the surface due to hydrodynamic trapping. However this happens only at a reduced distance of the surface (Frymier et al., 1995; Wood et al., 2006; Giacche et al., 2010). Besides, motility regulation overlaps with a complex signaling network that controls functions involved in biofilm formation such as quorum sensing or exopolysaccharide production (Merritt et al., 2007; Shrout et al., 2011). It has therefore been difficult to determine both a clear causal relationship and the mechanisms that could support a hypothesis of motility as an advantageous feature for biofilm-forming bacteria.

In *E. coli*, flagellar activity has been proposed to facilitate the initial contact of the cell with the surface, potentially helping to overcome repulsive forces on the surface (Pratt and Kolter, 1998). Nevertheless, when other surface appendages such as Curli or conjugative pili are constitutively expressed, flagella become dispensable for the initial adhesion and biofilm development (Prigent-Combaret et al., 2000; Reisner et al., 2003), suggesting motility *per se* might not be the adhesion promotion factor. On the other hand, flagella mechanosensory function as a surface-sensing tool has been proposed to govern the planktonic-sessile transition underlying biofilm formation (Laganenka et al., 2020; Wong et al., 2021). However, in this case the surface detection by the flagella culminates in motility downregulation. Consistently, high concentrations of the second messenger cyclic diguanylate (c-di-GMP) have been shown to correlate with motility downregulation and the development of a thick biofilm (Valentini and Filloux, 2016; Jenal et al., 2017). These results seem paradoxical with regard to the positive effect of motility on biofilm development, highlighting motility and biofilm development as mutually exclusive events. Nevertheless, bacteria may also swim within a mature biofilm (Houry et al., 2012).

Motility has also been suggested to influence biofilm maturation and architecture (Wood et al., 2006; Barken et al., 2008), although reports about the impact of cell motility on later stages of biofilm development are scarce. In *Vibrio cholerae*, motility has been proposed to favor the invasion of resident biofilms (Nadell et al., 2015). Ultimately, the motility effect on biofilm formation significantly changes depending on the environment, including surface properties and hydrodynamics (Zheng et al., 2021). Therefore, despite its obvious competitive fitness advantage in planktonic life (particularly regarding nutrient pursuit), the question of whether cell motility is a superior trait in surface-colonizing competition remains open.

To address this issue, we examined biofilm formation by motile and nonmotile *E. coli* cells in the controlled geometry of a millifluidic device from a kinetic perspective, covering both the short and long time scales of the adherent community development. This allowed us to search for mechanistic information that could distinguish the colonizing ability of swimming cells from their nonmotile counterparts. In this study, we take motility to mean flagellar motility apart from surface-associated motions such as swarming or twitching (Wadhwa and Berg, 2021). We thus examined the simple situation of motile vs. nonmotile *E. coli* strains colonizing a bare glass surface, followed by a more complex and more naturally relevant situation involving the same strains in the presence of other species in a co-colonization test. The latter included a 4-species assemblage that we previously showed to form a deterministic community in about 40 hours of growth under continuous nutrient flow in a millifluidic channel (Monmeyran et al., 2021). The use of this continuous flow growth mode makes it possible to control the physicochemical properties of the environment throughout the development of the biofilm, and ensures that the biofilms can be thoroughly compared.

Our results reveal a clear-cut effect of motility on surface colonization, which consists in introducing a lag time of several hours to the biofilm development. Meanwhile, the motile and nonmotile cells display a similar biofilm growth rate. We interpret this effect in terms of a spatial exploration discrepancy. Finally, we reveal how the presence of co-colonizers affects this behavior and discuss the strong dependence of flagellar motility effects on specific environmental conditions.

## Materials and Methods

### Bacterial Strains and Culture Conditions

*E. coli* strains were derived from *E. coli* K-12 classified as MG1655. In addition to the wild type motile strain, we used the nonmotile variant which lacks the insertion sequence IS*1* upstream of the *flh*D promoter. This consequently blocks cell swimming, as shown by flagellar motility tests (**Figure S1**). Both strains express the conjugative F-pilus carried by the F-plasmid IncFI, which ensures robust biofilm formation (Ghigo, 2001), and the FAST-mCherry fusion protein to provide biofilm-relevant fluorescence labeling (Monmeyran et al., 2018). For surface dynamics monitoring experiments, we used variants that constitutively express GFP. The co-colonizers belong to a previously described four-species community (Sheppard et al., 2013) consisting of *Bacillus thuringiensis* (*Bt*), a 407 Cry^-^ strain, *Pseudomonas fluorescens* (*Pf*) (WCS365), *Kocuria varians* (*Kv*) (CCL56), and *Rhodocyclus* sp. (*Rh*) (CCL5). For 4S biofilm kinetic monitoring, we used fluorescent *Bt* and *Pf* variants carrying the FAST gene on the chromosome (Monmeyran et al., 2021) and plasmidic mCherry (pMP7605) (Lagendijk et al., 2010), respectively. The strains were routinely cultivated at 30°C on M1 medium. Details about strains and culture media in **Supplementary Information**.

### Millifluidic Device

Millifluidic channels were microfabricated to be 30 mm x 1 mm x 1 mm (length x width x height). A polydimethylsiloxane (PDMS) mixture (RTV615A+B; Momentive Performance Materials) was poured at ambient temperature in a polyvinyl chloride home-micromachined mold and left to cure at least 3 hours in an oven set at 65°C. Next, the recovered templates were drilled for further plugging of adapted connectors and tubing. PDMS templates and glass coverslips were then cleaned using an oxygen plasma cleaner (Harrick) and immediately bound together to seal the channels. The channels are then immediately filled with ultrapure sterile water to avoid prolonged contact with air before injecting the cells within the following 3 to 4 hours. For connections, we used stainless steel connectors (0.013” ID and 0.025” OD) and microbore Tygon tubing (0.020” ID and 0.06” OD) supplied by Phymep (France). The thin metallic connectors provide a bottleneck in the flow circuit, which prevents upstream colonization. The sterile medium was pushed into the channels at a rate of 1 ml/h with syringe pumps for the 36-40 hours of the experiment. The whole experiment was thermostatically maintained at 30°C.

### Biofilm Formation

*Initiation:* 1.2×10^5^ cells were obtained from exponentially growing cultures. Cell injections were performed directly into the PDMS channels using a syringe equipped with a 22G needle before connecting the tubing. Next, the cells were allowed to settle for 1h30 before starting the medium flow. All times at t=0 referred to the flow triggering time. For biofilm growth, we used MB medium, which is adapted from M1 medium (details provided in **Supplementary Information**). Overnight cultures in M1 — seeded with a single colony from M1-agar plates — were grown at 30°C under agitation. Exponential phases were obtained from dilutions in M1 of these overnight cultures incubated at 30°C under agitation. The same protocol was applied in the presence of co-colonizers, except that 1.2×10^5^ cells from exponentially growing cultures of each co-colonizer were injected at the same time as *E. coli* into the channel.

### Microscope Imaging

*Microscopy:* We used an inverted NIKON TE300 microscope equipped with motorized x, y, and z displacements and shutters. Images were collected using a 20 × S plan Fluor objective (NA 0.45 WD 8.2-6.9 mm). Bright-field images were collected in direct illumination (no phase). Fluorescence acquisitions were performed using either the green channel filters for GFP and FAST : HBR-2,5-DM (Ex. 482/35, DM 506 Em. FF01-536/40) or the red filter for m-Cherry (Ex 562/40nm DM 593 Em. 641/75). Excitation was performed using an LED box (CoolLed pE-4000). For dynamics measurements, confocal images were collected using a spinning disk Crest X light V2 module (Gataca, France distribution) with an axial resolution of 5.8 µm.

### Image Acquisition

A Hamamatsu ORCA-R2 EMCCD camera was used for time-lapse acquisitions of 1344×1024 pixel images with 12-bit grey level depth (4096 grey levels), and to capture an *xy* field of view of 330 µm × 430 µm. Bright-field and fluorescence images were typically collected for 36 hours at a frequency of 6 frames per hour. Excitation LEDs were set at a 50% power level, and exposure times were 50 ms or 500 ms for the green emissions (for GFP and FAST, respectively) and 800 ms for the red emissions.

### Image Analysis

*Image intensities:* Time-lapse images were analyzed to derive the kinetics of *E. coli* biomass accumulation in the channel based on FAST fluorescence intensity, as previously detailed (Monmeyran et al., 2018). Image intensity per pixel, averaged for the whole image or on defined regions of interest (ROIs), was collected using the NIKON proprietary software NIS. Subsequently, the data sheets edited by NIS were exported to MATLAB for further analysis of biofilm development kinetics. Background was subtracted using the contribution to the fluorescence intensity of a channel containing medium without any bacteria (details in **Figure S2**). All curves were averaged over at least three independent positions and two independent replicates.

### Dynamics Monitoring

The individual dynamics of *E. coli* cells were tracked in the biofilm surface layer using confocal time-lapse acquisitions performed with a 60x, 1.4NA objective. Series of 50 images were recorded at an acquisition frequency of 90 frames per hour every 2 hours, for 24 h. The image stacks were binarized using the ImageJ IsoData threshold calculation tool, which iteratively takes into account average background and average object intensities. Next, objects comprising between 40-2,000 pixels in area (1px=0.1075 µm) were tracked in 2D over each temporal series using the ImageJ MTrack2 algorithm. These size limits ensured the tracking of a single cell to clusters of a few (3-4) cells. The minimal trajectory held 2 frames and the maximal accepted displacement between two frames was 60 pixels. Thereafter, the results file containing the sorted coordinates of all the trajectories was exported to MATLAB in order to calculate the trajectory persistence *P*. For each time series, *P* was obtained by taking the average over all the trajectories *i* of the individual persistence *P_i_*, defined as the ratio of the travelled distance *d_i_* to the trajectory length *L_i_*.

## Results

### Motility Strongly Delays Bare Surface Colonization by *E. coli*

**Figure 1:**
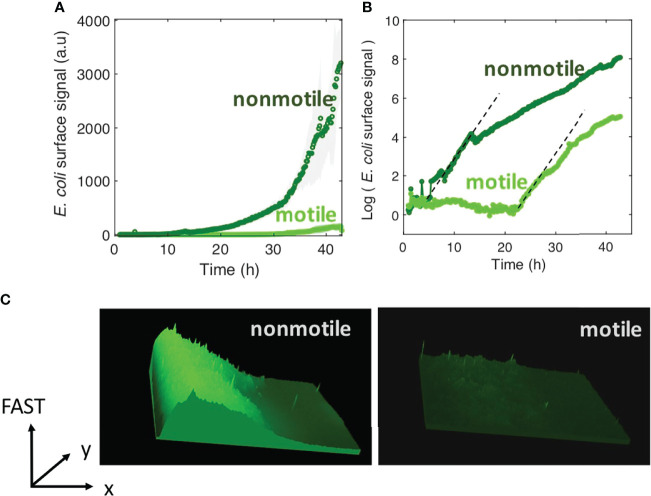
Motility delays biofilm formation. **(A)** *E. coli* FAST fluorescence intensity as a function of flow time for a biofilm grown by motile (bright green) and nonmotile (dark green) cells in a millifluidic channel; bold curves are from the average intensity of 3 distinct positions in 2 or 3 different channels with the shaded area representsing the standard deviation **(B)** Same data as in A except the logarithm of the average fluorescent intensity is plotted. The biofilm was grown at a rate of 1 ml/h medium flow, at 30°C. Dashed lines highlight the exponential part of the growth and display the same slope for both motile and nonmotile cells. **(C)** Fluorescence intensity surface plots from biofilm images recorded at time t=30h. Biofilm from nonmotile cells on the left panel and from motile on the right panel.

**Figure 2:**
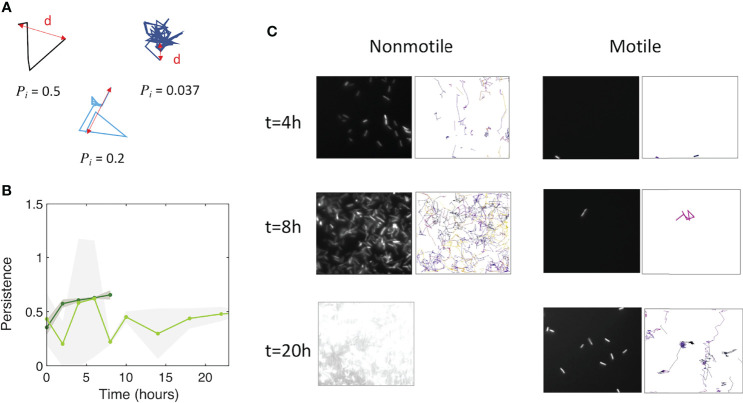
Motile and nonmotile cells display similar surface persistence in the biofilm initiation phase. **(A)** Typical trajectories with their respective persistence index values. **(B)** Persistence values over time for motile (light green) and nonmotile (dark green) cells on the surface. The curves represent the average, with the shaded area representing the standard deviation. **(C)** Snapshots of the biofilm development together with detected trajectories for nonmotile (left columns) and motile cells (right columns). At t=20h, the surface of nonmotile cells was overcrowded, which excludes the ability to track single cells.

We assessed how fast the bacteria form a biofilm under mild flow depending on their motility by comparing the surface colonization kinetics of the nonmotile cells (Mot^-^) with that of their swimming counterparts (Mot^+^). Time-lapse images were taken from the very beginning of the process, consisting of a few adhering cells, up to the stage of a dense cell material after 48 hours of growth. The results show very distinct kinetic profiles for motile and nonmotile cells (**Figure 1A**). Using a logarithm scale to display the fluorescence intensity as a function of time revealed that this difference mainly consists in a long lag time of about 20 hours, whereas almost no lag time was evident for nonmotile cells (**Figure 1B**). In contrast, the colonization rate measured after the lag appears very similar for motile and nonmotile bacteria, indicating that the biofilm initiation phase was essentially altered when cells were motile as opposed to nonmotile. Furthermore, we measured the cell division rates under planktonic growth in MB medium and observed no differences (**Figure S3**). To determine the details of this initial phase, we recorded stacks of images at a higher frequency (90 images per hour instead of 6) every two hours, using a higher magnification objective (63x) and confocal acquisitions in order to make observations at the single cell level and evaluate the surface dynamics. Applying a basic 2D tracking routine to this image series, we collected cell trajectories for each time series and derived a persistence index, *P*. The mean of all individual trajectory *P_i_* values was defined as the ratio of the trajectory length *L_i_* to the traveled distance *d_i_* as shown in **Figure 2A**. This index provides a quantitative evaluation of the cell dynamics on the surface that varies from 0 for a fully steady cell to 1, which corresponds to the highest displacement in a straight line authorized in the analysis, i.e. the strongest dynamics. The analysis concentrates on the initial phase of the colonization where cell population is limited enough to enable single cell delineation, corresponding to the first 6 hours of biofilm formation for nonmotile cells and extending up to 24 hours for motile cells. The results in **Figure 2B** show the variation of the dynamic index for motile and nonmotile cells over time. Due to surface access delay, the number of trajectories included in the motile cell analysis within the first 10 hours is low (5 to 10 per time series compared to 50 to100 for nonmotile cells, see **Figure S4**) causing large standard deviations and significant fluctuations of the mean values on this data set which complicated the comparison with the nonmotile cells. However, comparing equivalent colonization degrees as for time t=4h (nonmotile) and time t=20h (motile) shown in **Figure 2C**, very close persistence values were obtained confirming the similar dynamics on the surface of the two cells type. The small number of cells on the surface also explains the high observed standard deviation of the persistence of motile cells.

**Figure 3:**
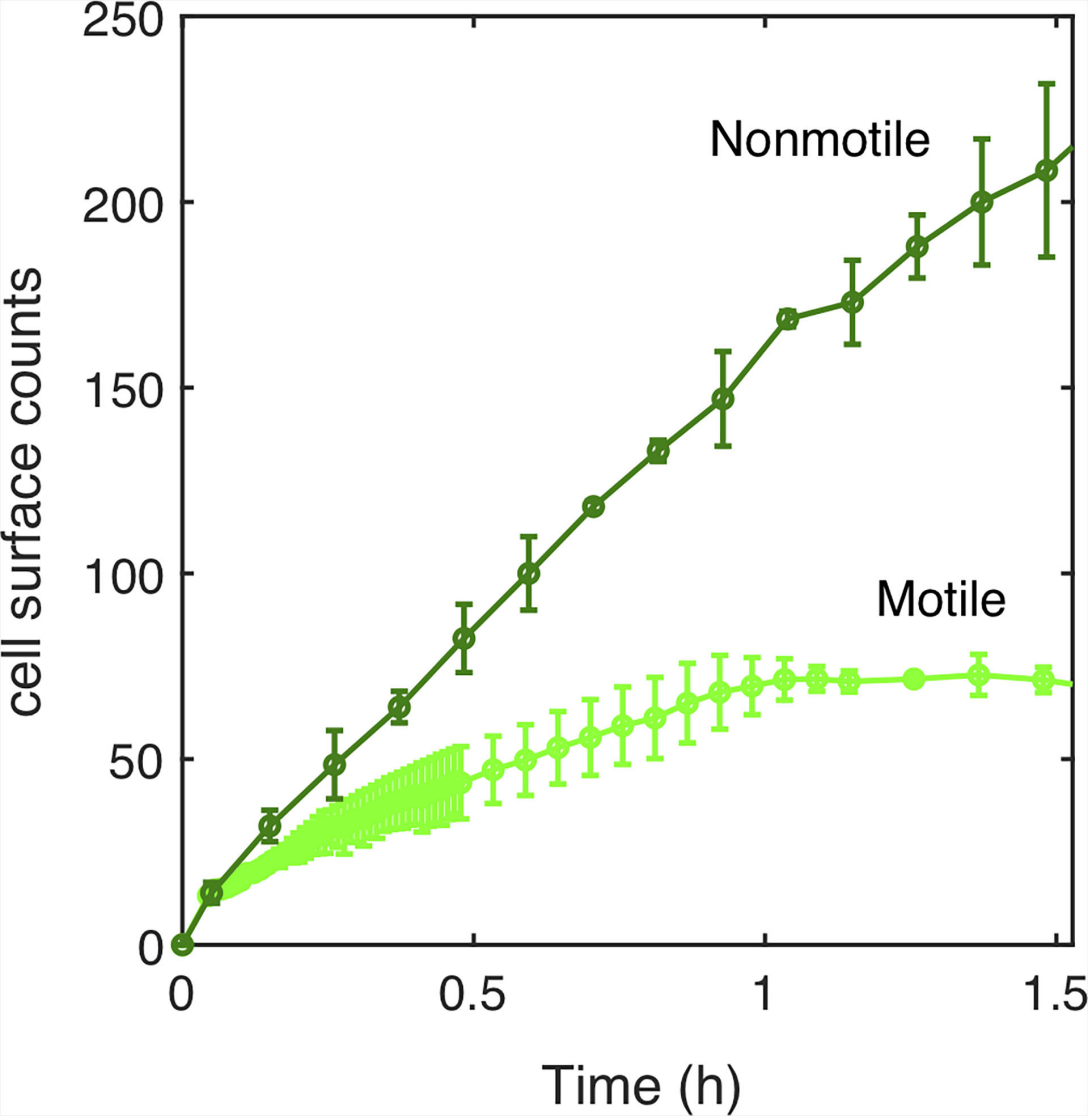
Surface access experimental kinetics of nonmotile and motile cells. The number of cells reaching the surface over the incubation time (90 min) has been determined experimentally for motile (light green curve) and nonmotile (dark green curve). 1.6x10^6^ cells/ml were injected in MB medium at time t=0. The surface was regularly imaged to count the settled cells. Errors bars are standard deviation over 3 different positions.

The fraction of cells reaching the surface, and not their dynamics on the surface, is therefore more likely to explain the lingering of motile cells during biofilm initiation as compared to Mot^-^ cells. To test this idea, we measured the number of motile and nonmotile cells reaching the surface over the first 90 min following the injection in the channel before starting the flow and actually found that there were fewer motile cells (**Figure 3**). Then, by making a reasonable hypothesis about biofilm development kinetics following a logistic law that closely fits the data (**Supplementary Information; Figure S5**), we found that the motile-nonmotile cell asymmetry in the surface abundance explained the observed significant delay in biofilm growth. Indeed, as flow begins, the non-attached cells are removed from the channel and no longer participate in biofilm formation.

To examine how cell bulk behavior could explain the motile cells colonization deficit. A simple theoretical model was created to evaluate motile versus nonmotile cell behavior.

### Motile and Nonmotile Cells Characteristic Time for Cell-Surface Access

In our model, we considered a bacterial suspension in a box (defined by volume *V* and height *H*) and we wrote equations to describe the variation with time for the number of cells reaching the bottom surface upon settling (nonmotile cells) as well as upon settling and run-and-tumble diffusion (motile cells). As detailed in the **Supplementary Information**, cell settling occurred according to the unidirectional speed V_s_ (Stokes’ law) of a particle submitted to gravity in a viscous medium. This therefore provides a number of cells $N_{s}^{g}$ on the surface as a function of time (for *t* < *H*/*V_S_*):


$$N_{s}^{g}\left(t\right)=\frac{N_{0}}{H}V_{S}t$$

(For *t* > *H*/*V_S_*, all cells have reached the bottom surface and $N_{s}^{g}\left(t\right)=N_{0}.)$

For diffusing cells, we made numerical simulations to account for both the settling and the diffusion, deriving the number of cells to an absorbing boundary. A number N_0_ of cells were randomly distributed along a 1D interval [0,H]. Every period of time dt (dt=1s), each cell could jump up or down with equal probability (1/2), by a step $dz=\sqrt{2Dt}$ with D, the cell diffusion coefficient. In addition, all cells were moved down by a distance *V_S_*.*dt* to account for the sedimentation. All cells that moved beyond the upper limit (*z* > *H*) were brought back to *z* = *H*. This reflective boundary condition accounts for the experimental observations which showed no attached cell on the top surface within the first hours following the cell injection in the channel. All cells that moved below the lower limit (*z* < 0) were removed from the simulation (absorbing boundary condition, first passage attachment) and counted as “surface cells”, $N_{s}^{d}\left(t\right)$ .

**Figure 4:**
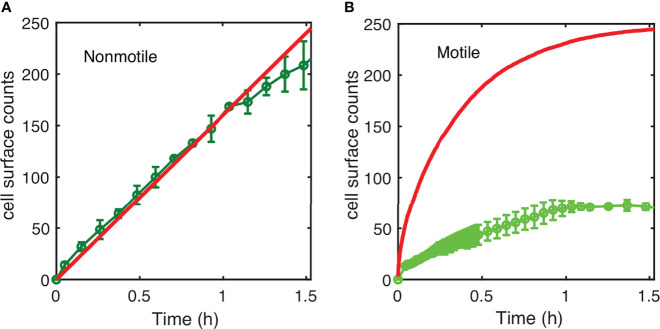
Simulated kinetics of settling and diffusion towards the surface. **(A)** The number of nonmotile cells reaching the surface as a function of time in a 1-mm high channel was calculated according to settling using the diffusion coefficient “D=10 µm^2^/s” and “D=200 µm^2^/s” cell size a=1 µm; g=10 m/s^2^; and cell-medium mass density contrast Δ*ρ* = 80 *kg*/*m* ^3^ (red line) and compared to the experimental counts (same as in **Figure 3**). **(B)** Numerical simulation of surface access kinetics of motile cells (red line) represented with the corresponding experimental counts (as in **Figure 3**).

The results displayed in **Figure 4** show the simulation results compared to the experimental counts for both nonmotile (**Figure 4A**) and motile cells (**Figure 4B**) using a diffusion coefficient of 200 µm^2^/s (**Figure S6**) in agreement with previous work e.g. (Wu et al., 2006). The model satisfyingly predicts the experimental surface access kinetics of the nonmotile cells, which was confirmed by measurements and calculations performed using a channel with 250 *µ*m of height (**Supplementary Information; Figure S7**). In contrast, the motile cell behavior was not correctly described by the model which predicts a much faster surface access than the experiments (**Figure 4B**). Indeed, the predicted cell number was about five times higher in the simulation than in the experimental counts. This suggests that the hypothesis of attachment at first passage is probably too restrictive and that the swimming cells may experiment several passages before dwelling onto the surface. On the other hand, the motile cell diffusion might be biased due to the topology of the device. Indeed, because of the asymmetry of the PDMS channels, oxygen gradients that would favor bacteria swimming upwards, could have appeared delaying the surface access.

Altogether, these results indicate that surface access is the limiting step of the process. The simple mathematical model we introduce here suggests that under the conditions used in this study, the nonmotile settle and attach whereas the motile cells swim away from the surface beyond the predictions of a random diffusion and first passage attachment hypothesis.

### The Presence of Co-Colonizers Affects the Surface Colonization Process for Both Motile and Nonmotile Cells

In order to evaluate if cell motility brings about a similar lag time in a more complex environment, we monitored how motile and nonmotile *E. coli* cells colonize a bare surface in the presence of co-colonizers. For this, we examined surface colonization by *E. coli* in the presence of four other strains that we recently showed are able to deterministically form a stable biofilm under flow in about 40 hours (Monmeyran et al., 2021).

**Figure 5:**
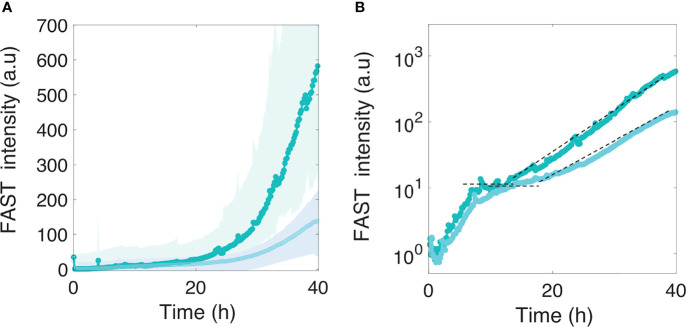
Motile and nonmotile *E. coli* surface colonization in the presence of co-colonizers. Motile (in light cyan) and nonmotile (in dark cyan) *E. coli* surface fluorescence **(A)** in the biofilm formed in the presence of the four co-colonizers *Bt, Pf, Kv* and *Rh*. The logarithmic display **(B)** highlights two distinct phases in *E. coli* development in the composite multispecies biofilm. The dashed lines indicate the level of the first phase plateau and the second phase growth rate. Curves are from the average intensity of 3 distinct positions from 2 different channels (2 biological replicates) with the shaded area representing the standard deviation.

The four species were introduced in the channel at the same time as the motile or nonmotile *E. coli* cells, following the same procedure used for single species biofilm formation and kinetics monitoring. The results in **Figure 5A** show that motile bacteria still display a slight deficit in colonization efficiency, although the difference between motile and nonmotile cells was significantly reduced in the presence of the co-colonizers. Notably, the presence of the co-colonizers altered not only the amplitude but also the kinetic phases of development, as highlighted by the logarithmic display of the curves (**Figure 5B**). In the presence of the co-colonizers, we observed the succession of two phases displaying the same timing for motile and nonmotile bacteria. The first phase consisted of an initial limited surface growth that leveled about 6 hours after starting the nutrient flow, with a plateau that extended approximately up to 12 to 15 hours into colonization. Subsequently, a second growth phase started which increased the *E. coli* biomass in the composite biofilm by 2 logs. The curves indicate that the motile cell biofilm entered the second growth phase with a small 2- to 3-hour delay.

**Figure 6:**
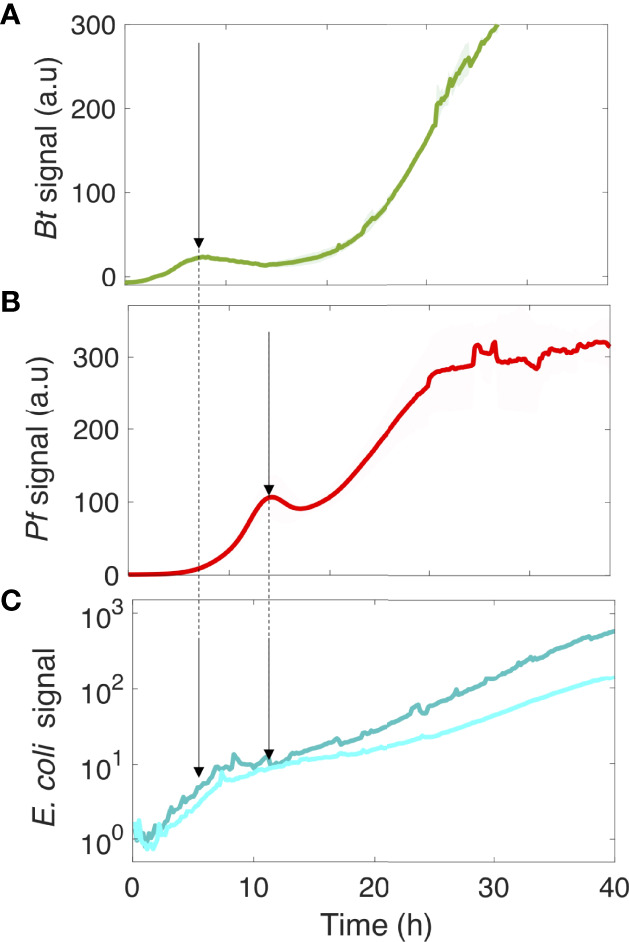
The kinetics of motile and nonmotile *E. coli* surface colonization follows the community development timing of the four species. *Bt* **(A)** and *Pf* **(B)** surface development kinetics in the four-species community. In parallel, the logarithmic display of the *E. coli* signal on the surface is shown in the presence of the co-colonizers **(C)**. The arrows point to the *Bt* **(A)** and *Pf* **(B)** initial climaxes, and the *E. coli* development phases **(C)**. Curves are from the average intensity of 2 distinct positions in 3 different channels (3 biological replicates) with the shaded area representing the standard deviation.

We next asked how these kinetics could be related to the development of the biofilm colonizers. To answer this question, we monitored the development of the four-species biofilm injecting the fluorescent variants of *Bacillus thuringiensis* (*Bt-*FAST) and *Pseudomonas fluorescens* (*Pf*-mCherry), the two main contributors in the four-species community, with *Kocuria varians* and *Rhodocyclus* which were not labelled. **Figures 6A, B** specifically shows the kinetics of these two fluorescent species in the four-species biofilm.

The saturation of *E. coli* development in its initial phase coincided with the first climax of *Bt* in the four-species community, whereas the second growth phase of *E. coli* started as *Pf* development reached its first climax. These results indicate that the deterministic biofilm formation program of the co-colonizers dominated the *E. coli* surface colonization process, inducing a kinetic remodeling and almost completely abolishing the difference between motile and nonmotile cells in their colonization efficiency. This indicates that the limiting step of the surface colonization by *E. coli* is shifted by the presence of the co-colonizers, significantly altering the impact of motility in the process.

Finally, we also tested the colonization of the pre-established four-species biofilm by the motile and nonmotile strains of *E. coli* at 8, 20 and 36 hours after inoculation and incubation of the four species. This demonstrated that the corresponding signals barely emerged from the background for both motile and nonmotile cells 40 hours after their injection (**Supplementary Information; Figure S8**), indicating that neither of the two strains were able to colonize the surface in the presence of the pre-established strains.

## Discussion

Bacterial flagellar motility has been investigated for decades now, and significant advances in the understanding of the involved mechanisms have been made. However, many open questions remain about the impact of this function on the ability of bacteria to colonize surfaces. Here, we report a kinetic analysis of *E. coli* surface colonization in a millifluidic channel, with a focus on active vs. non-active flagellar motility. Our results show that motility introduces a significant delay to the colonization of bare surfaces by *E. coli* alone. This is in contrast to the intuitive concept encountered in the literature that the absence of motility reduces the chances of bacterial cells coming into contact with the surface (Pratt and Kolter, 1998; Zheng et al., 2021). Based on mathematical modeling of the settling and diffusion of the bacteria in the channel, we found that the nonmotile cells landed on surface following a simple settling law. In contrast, random diffusion did not account for motile cell on surface over time. In our experimental configuration, the ‘race’ towards the surface is won by nonmotile cells which is not predicted by the calculations. To explain this discrepancy, we suggest that motile cells may repeatedly bounce on the surface before attaching which would slow down the dwelling kinetic. Besides, taking into account the asymmetry of the experimental channel, we make the hypothesis that chemical gradients may also bias bacteria swimming and delay surface colonization.

The channel geometry that we used is relevant for many natural situations in which bacteria dwell in channels and pores in the millimetric range under continuous or intermittent flow with irregular nutrient spatial distribution and chemical gradients. This highlights the importance of the geometry of the environment, which has generally been overlooked in colonization assays, in the competition between motile and nonmotile cells.

We observed that local dynamics and biomass growth rates were very similar for both motile and nonmotile cells, suggesting that flagellar motility does not alter anchorage or surface persistence once the cells have reached the surface, an issue that has remained controversial to date. As detailed in previous investigations of this question (Pratt and Kolter, 1998), it is difficult to decipher the primary factors involved in the initial attachment. A particular challenge is to disentangle the contribution of motility *per se* from the contribution of flagella as a surface appendage and the contingent involvement of other structures such as type I pili. Under the conditions used in this study for bare surface colonization, it is likely that the attachment step is dominated by the overexpression of the F-pilus, which is crucial in promoting the initial adhesion (Ghigo, 2001; Reisner et al., 2003; Beloin et al., 2008). In this case, flagellar motility simply reduces surface abundance, which is established during the inoculation period, and does not affect biofilm development. This is an interesting finding to take into account for multispecies colonization processes where surface access kinetics are crucial in the competitive dynamics that shape the attached community (Eigentler et al., 2022).

In the presence of co-colonizers (here, the members of a 4-species community able to build a stable biofilm), we observed that motile and nonmotile *E. coli* cells exhibit very similar colonization profiles that differ from the profiles displayed in the single-species colonization experiments. Specifically, there is a lower surface-bound *E. coli* global biomass, consistent with the intrinsic competition for the surface expected from the presence of other adhesive species (Lloyd and Allen, 2015). Two striking features stand out here: the emergence of *E. coli* kinetic colonization phases that match the four-species biofilm climaxes; and the reduced lag observed between motile and nonmotile cells in the characteristic time of colonization, primarily due to the receding of the nonmotile cells in comparison to the *E. coli* colonizing ability in the absence of co-colonizers.

In a previous study, the four-species biofilm climaxes were interpreted as species responses to the oxygen depletion induced by biofilm development, suggesting that the reduction in oxygen might also contribute to the diminished colonizing efficiency of *E. coli (* Monmeyran et al., 2021). Moreover, knowing that the lack of oxygen strongly affects *E. coli* motility (Douarche et al., 2009) by inducing a motile to nonmotile transition in the bacterial population, we can hypothesize that the environmental oxygen scarcity caused by the co-colonizers accounts for the convergence of the motile and nonmotile cell colonization kinetics in this multispecies context. Nevertheless, the co-colonizers could also induce a shift in the limiting step of the colonizing process by increasing the characteristic time of attachment on the surface, which would result in abolishing the difference between motile and nonmotile cells that ultimately dwell on the surface. These results stress the importance of studying the processes from a kinetic perspective in order to acquire mechanistic information.

Our report establishes that the impact of flagellar motility on surface colonization is not necessarily an intrinsic trait associated with this function; instead, it closely depends on an environment defined by both topology and population composition. Importantly, we demonstrate that cell swimming can regulate the surface access time. However, a shift in the environmental conditions (such as the presence of co-colonizers) can drastically alter the outcome of this distinctive property and abolish the asymmetry between motile and nonmotile cells. We thus propose here a study with the potential to enlighten the long-standing controversy over the role of cell motility in surface colonization and biofilm formation.

## Data Availability Statement

The original contributions presented in the study are included in the article/**Supplementary Material**. Further inquiries can be directed to the corresponding author.

## Author Contributions

WBY performed the experiments and analyzed the data. MD developed the mathematical model and reviewed the manuscript. AM initiated experiments. NH designed the study, analyzed the data and wrote the paper. All authors contributed to the article and approved the submitted version.

## Funding

This work was supported by a grant from the French Agence Nationale pour la Recherche (ANR- 15-CE02-0001-01 ACToP), and a MESRI fellowship to WBY.

## Conflict of Interest

The authors declare that the research was conducted in the absence of any commercial or financial relationships that could be construed as a potential conflict of interest.

## Publisher’s Note

All claims expressed in this article are solely those of the authors and do not necessarily represent those of their affiliated organizations, or those of the publisher, the editors and the reviewers. Any product that may be evaluated in this article, or claim that may be made by its manufacturer, is not guaranteed or endorsed by the publisher.

## References

[B1] Barken K. B. Pamp S. J. Yang L. Gjermansen M. Bertrand J. J. Klausen M.. (2008). Roles of Type IV Pili, Flagellum-Mediated Motility and Extracellular DNA in the Formation of Mature Multicellular Structures in Pseudomonas Aeruginosa Biofilms. Environ. Microbiol. 10, 2331–2343. doi: 10.1111/j.1462-2920.2008.01658.x 18485000

[B2] Beloin C. Houry A. Froment M. Ghigo J. M. Henry N. (2008). A Short-Time Scale Colloidal System Reveals Early Bacterial Adhesion Dynamics. PloS Biol. 6, e167. doi: 10.1371/journal.pbio.0060167 18613749 PMC2443189

[B3] Berg H. C. Anderson R. A. (1973). Bacteria Swim by Rotating Their Flagellar Filaments. Nature 245, 380–382. doi: 10.1038/245380a0 4593496

[B4] Colin R. Ni B. Laganenka L. Sourjik V. (2021). Multiple Functions of Flagellar Motility and Chemotaxis in Bacterial Physiology. FEMS Microbiol. Rev 6, 1–19. doi: 10.1093/femsre/fuab038 PMC8632791 34227665

[B5] Douarche C. Buguin A. Salman H. Libchaber A. E. (2009). Coli and Oxygen: A Motility Transition. Phys. Rev. Lett. 102, 198101. doi: 10.1103/PhysRevLett.102.198101 19518998

[B6] Eigentler L. Kalamara M. Ball G. MacPhee C. E. Stanley-Wall N. R. Davidson F. A.. (2022). Founder Cell Configuration Drives Competitive Outcome Within Colony Biofilms. ISME. J 16, 1512–22. doi: 10.1038/s41396-022-01198-8 PMC9122948 35121821

[B7] Flemming H. C. Wingende J. Szewzyk U. Steinberg P. Rice S. A. Kjelleberg S. (2016). Biofilms: An Emergent Form of Bacterial Life. Nat. Rev. Microbiol. 14, 563–575. doi: 10.1038/nrmicro.2016.94 27510863

[B8] Frymier P. D. Ford R. M. Berg H. C. Cummings P. T. (1995). Three-Dimensional Tracking of Motile Bacteria Near a Solid Planar Surface. Proc. Natl. Acad. Sci. U.S.A. 92, 6195–6199. doi: 10.1073/pnas.92.13.6195 7597100 PMC41669

[B9] Ghigo J. M. (2001). Natural Conjugative Plasmids Induce Bacterial Biofilm Development. Nature 412, 442–445. doi: 10.1038/35086581 11473319

[B10] Giacche D. Ishikawa T. Yamaguchi T. (2010). Hydrodynamic Entrapment of Bacteria Swimming Near a Solid Surface. Phys. Rev. E. Stat. Nonlin. Soft. Mat. Phys. 82, 56309. doi: 10.1103/PhysRevE.82.056309 21230578

[B11] Haiko J. Westerlund-Wikstrom B. (2013). The Role of the Bacterial Flagellum in Adhesion and Virulence. Biol. (Basel). 2, 1242–1267. doi: 10.3390/biology2041242 PMC4009794 24833223

[B12] Hall-Stoodley L. Costerton J. W. Stoodley P. (2004). Bacterial Biofilms: From the Natural Environment to Infectious Diseases. Nat. Rev. Microbiol. 2, 95–108. doi: 10.1038/nrmicro821 15040259

[B13] Houry A. Gohar M. Deschamps J. Tischenko E. Aymerich S. Gruss A.. (2012). Bacterial Swimmers That Infiltrate and Take Over the Biofilm Matrix. Proc. Natl. Acad. Sci. U.S.A. 109, 13088–13093. doi: 10.1073/pnas.1200791109 22773813 PMC3420162

[B14] Jenal U. Reinders A. Lori C. (2017). Cyclic Di-GMP: Second Messenger Extraordinaire. Nat. Rev. Microbiol. 15, 271–284. doi: 10.1038/nrmicro.2016.190 28163311

[B15] Josenhans C. Suerbaum S. (2002). The Role of Motility as a Virulence Factor in Bacteria. Int. J. Med. Microbiol. 291, 605–614. doi: 10.1078/1438-4221-00173 12008914

[B16] Karygianni L. Ren Z. Koo H. Thurnheer T. (2020). Biofilm Matrixome: Extracellular Components in Structured Microbial Communities. Trends Microbiol. 28, 668–681. doi: 10.1016/j.tim.2020.03.016 32663461

[B17] Laganenka L. Lopez M. E. Colin R. Sourjik V. (2020). Flagellum-Mediated Mechanosensing and RflP Control Motility State of Pathogenic Escherichia Coli. mBio 11, 1–11. doi: 10.1128/mBio.02269-19 PMC7157525 32209689

[B18] Lagendijk E. L. Validov S. Lamers G. E. de Weert S. Bloemberg G. V. (2010). Genetic Tools for Tagging Gram-Negative Bacteria With Mcherry for Visualization *In Vitro* and in Natural Habitats, Biofilm and Pathogenicity Studies. FEMS Microbiol. Lett. 305, 81–90. doi: 10.1111/j.1574-6968.2010.01916.x 20180857

[B19] Lemon K. P. Higgins D. E. Kolter R. (2007). Flagellar Motility is Critical for Listeria Monocytogenes Biofilm Formation. J. Bacteriol. 189, 4418–4424. doi: 10.1128/JB.01967-06 17416647 PMC1913361

[B20] Lloyd D. P. Allen R. J. (2015). Competition for Space During Bacterial Colonization of a Surface. J. R. Soc. Interface 12, 608. doi: 10.1098/rsif.2015.0608 PMC4614474 26333814

[B21] Merritt P. M. Danhorn T. Fuqua C. (2007). Motility and Chemotaxis in Agrobacterium Tumefaciens Surface Attachment and Biofilm Formation. J. Bacteriol. 189, 8005–8014. doi: 10.1128/JB.00566-07 17766409 PMC2168663

[B22] Monmeyran A. Thomen P. Jonquiere H. Sureau F. Li C. Plamont M. A.. (2018). The Inducible Chemical-Genetic Fluorescent Marker FAST Outperforms Classical Fluorescent Proteins in the Quantitative Reporting of Bacterial Biofilm Dynamics. Sci. Rep. 8, 10336. doi: 10.1038/s41598-018-28643-z 29985417 PMC6037777

[B23] Monmeyran A.. (2021). Four Species of Bacteria Deterministically Assemble to Form a Stable Biofilm in a Millifluidic Channel. NPJ Biofilms. Microbio. 7, 64. doi: 10.1038/s41522-021-00233-4 PMC8342524 34354076

[B24] Nadell C. D. Drescher K. Wingreen N. S. Bassler B. L. (2015). Extracellular Matrix Structure Governs Invasion Resistance in Bacterial Biofilms. ISME. J. 9, 1700–1709. doi: 10.1038/ismej.2014.246 25603396 PMC4511925

[B25] Nakamura S. Minamino T. (2019). Flagella-Driven Motility of Bacteria. Biomolecules 9, 1–23. doi: 10.3390/biom9070279 PMC6680979 31337100

[B26] O’Toole G. A. Kolter R. (1998). Flagellar and Twitching Motility are Necessary for Pseudomonas Aeruginosa Biofilm Development. Mol. Microbiol. 30, 295–304. doi: 10.1046/j.1365-2958.1998.01062.x 9791175

[B27] Pratt L. A. Kolter R. (1998). Genetic Analysis of Escherichia Coli Biofilm Formation: Roles of Flagella, Motility, Chemotaxis and Type I Pili. Mol. Microbiol. 30, 285–293. doi: 10.1046/j.1365-2958.1998.01061.x 9791174

[B28] Prigent-Combaret C. Prigent-Combaret C. Prensier G. Le Thi T. T. Vidal O. Lejeune P.. (2000). Developmental Pathway for Biofilm Formation in Curli-Producing *Escherichia Coli* Strains: Role of Flagella, Curli and Colanic Acid. Environ. Microbiol. 2, 450–464. doi: 10.1046/j.1462-2920.2000.00128.x 11234933

[B29] Reisner A. Haagensen J. A. Schembri M. A. Zechner E. L. Molin S. (2003). Development and Maturation of *Escherichia Coli* K-12 Biofilms. Mol. Microbiol. 48, 933–946. doi: 10.1046/j.1365-2958.2003.03490.x 12753187

[B30] Sheppard A. E. Poehlein A. Rosenstiel P. Liesegang H. Schulenburg H. (2013). Complete Genome Sequence of Bacillus Thuringiensis Strain 407 Cry. Genome Announc. 1, e00691–17. doi: 10.1128/genomeA.00158-12 23405326 PMC3569317

[B31] Shrout J. D. Tolker-Nielsen T. Givskov M. Parsek M. R. (2011). The Contribution of Cell-Cell Signaling and Motility to Bacterial Biofilm Formation. MRS. Bull. 36, 367–373. doi: 10.1557/mrs.2011.67 22053126 PMC3204577

[B32] Silverman M. Simon M. (1974). Flagellar Rotation and the Mechanism of Bacterial Motility. Nature 249, 73–74. doi: 10.1038/249073a0 4598030

[B33] Suchanek V. M.. (2020). Chemotaxis and Cyclic-Di-GMP Signalling Control Surface Attachment of Escherichia Coli. Mol. Microbiol. 113, 728–739. doi: 10.1111/mmi.14438 31793092

[B34] Valentini M. Filloux A. (2016). Biofilms and Cyclic Di-GMP (C-Di-GMP) Signaling: Lessons From Pseudomonas Aeruginosa and Other Bacteria. J. Biol. Chem. 291, 12547–12555. doi: 10.1074/jbc.R115.711507 27129226 PMC4933438

[B35] Wadhwa N. Berg H. C. (2021). Bacterial Motility: Machinery and Mechanisms. Nat. Rev. Microbiol 20, 161–73. doi: 10.1038/s41579-021-00626-4 34548639

[B36] Wong G. C. L. Antani J. D Lele P. P Chen J. Nan B. Kühn M. J. (2021). Roadmap on Emerging Concepts in the Physical Biology of Bacterial Biofilms: From Surface Sensing to Community Formation. Phys. Biol. 18, 1–49. doi: 10.1088/1478-3975/abdc0e PMC8506656 33462162

[B37] Wood T. K. Gonzalez Barrios A. F. Herzberg M. Lee J. (2006). Motility Influences Biofilm Architecture in Escherichia Coli. Appl. Microbiol. Biotechnol. 72, 361–367. doi: 10.1007/s00253-005-0263-8 16397770

[B38] Wu M. Roberts J. W. Kim S. Koch D. L. DeLisa M. P. (2006). Collective Bacterial Dynamics Revealed Using a Three-Dimensional Population-Scale Defocused Particle Tracking Technique. Appl. Environ. Microbiol. 72, 4987–4994. doi: 10.1128/AEM.00158-06 16820497 PMC1489374

[B39] Zheng S. Zheng S. Bawazir M. Dhall A. Kim H. E. He L.Heo J.. (2021). Implication of Surface Properties, Bacterial Motility, and Hydrodynamic Conditions on Bacterial Surface Sensing and Their Initial Adhesion. Front. Bioeng. Biotechnol. 9. doi: 10.3389/fbioe.2021.643722 PMC7907602 33644027

