# Supplementary material for: Flagellar Motility During E. coli Biofilm Formation Provides a Competitive Disadvantage Which Recedes in the Presence of Co-Colonizers
Source: Front Cell Infect Microbiol. 2022 Jul 8;12:896898. doi: 10.3389/fcimb.2022.896898 (PMC9307998; doi:10.3389/fcimb.2022.896898)
Supplement: Supplementary file 1 [file Presentation_1.pdf]

Supplementary information

I. Materials and methods

- Media composition

Composition	**M1** (g/L)	**MB** (g/L)
Yeast Nitrogen Base*	1.7	1.7
Ammonium sulfate	5	5
Glucose	10	0.4
Casamino-acids	5	1

*from DIFCO BD

- Strains and plasmids

strains		Plasmids and reporters	References
1	*E. coli* MG1655 F motile	F plasmid, IncF from TG1 (Tet^R)	Ghigo (2001)[1]
2	*E. coli* MG1655 nonmotile (IS*1*$^-$)	F plasmid[a] (Tet^R)	Ghigo (2001)[1]
5	*E. coli* MG1655 F motile – FAST-mCherry	F plasmid[a] (Tet^R)<br>pAG101d[b] (Kan^R)	Ghigo (2001)[1]<br>Plamont et al. (2016)[2]
6	*E. coli* MG1655 F nonmotile – FAST-mCherry (IS*1*$^-$)	F plasmid[a]<br>pAG101d[b] (Kan^R)	Ghigo (2001)[1]<br>Plamont et al. (2016)[2]
	E. coli MG1655 F motile - GFP	F plasmid[a]<br>λatt::*gfp*-mut3 lambda p*R* (Amp^R)	Ghigo (2001)[1]
	E. coli MG1655 F nonmotile (IS*1*)-GFP	F plasmid[a]<br>λatt::*gfp*-mut3 (Amp^R)	Ghigo (2001)[1]
5	*P. fluorescens* WCS365 m-Cherry	pMP7605	Lagendijk et al. (2010)[3]
4	B.thuringiensis FAST	PsarA-*yfast* (erythromycin^R)[c]	A gift from A. Baliarda

[a]as in strain #1 :IncF from TG1
[b]derived from pAG101 by replacing T7 promoter by Lambda p*R*
[c]*yfast* sequence from pAG86 inserted on the chromosome.

Strains references:

1 Ghigo, J. M. Natural conjugative plasmids induce bacterial biofilm development. *Nature* **412**, 442-445 (2001).

2 Plamont, M. A. *et al.* Small fluorescence-activating and absorption-shifting tag for tunable protein imaging in vivo. *Proc Natl Acad Sci U S A* **113**, 497-502,(2016).

3 Lagendijk, E. L., Validov, S., Lamers, G. E., de Weert, S. & Bloemberg, G. V. Genetic tools for tagging Gram-negative bacteria with mCherry for visualization in vitro and in natural habitats, biofilm and pathogenicity studies. *FEMS Microbiol Lett* **305**, 81-90, (2010).

- **Motility assay**

Motile and nonmotile strains swimming motility was tested according to an adaptation of the protocol of Barker and collaborators [4]. Aliquots of the same concentration (10^6cells/ml) of motile and nonmotile *E. coli* cells from overnight cultures were inoculated into a soft agar (LB semi-solidified with 1.25% wt/vol agar enabling flagellar motility [4]. The plates were incubated at 30°C during 24h and imaged. The results are shown in Fig. S1. The diffuse spreading of the motile cells can be easily recognized by eye in comparison with the small nonmotile cells colony where no outgrowth occurred.

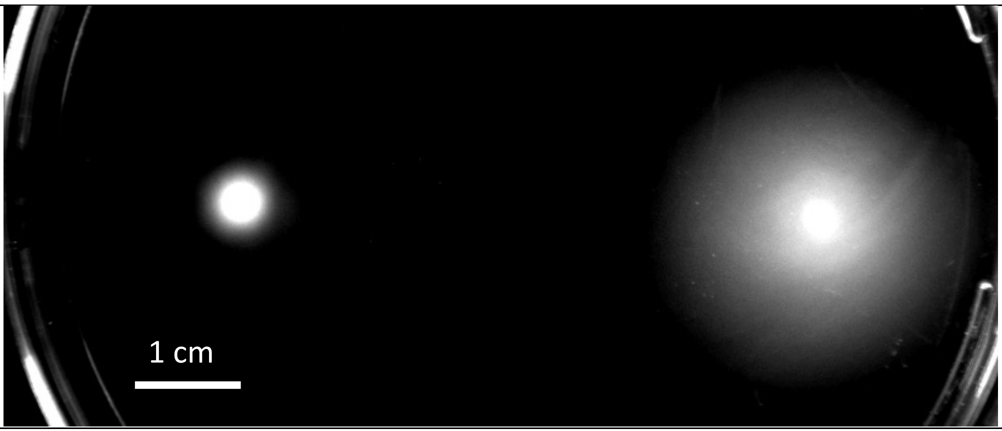

Figure S1: Motility assay. Image of nonmotile (on the left) and motile (on the right) cells inoculated in 1.25% wt/vol agar after 24 hours growth in MB medium.

- **Background versus signal amplitude**

E. coli signal on the surface is obtained is obtained by averaging FAST intensity over the whole image (1344x1024 pixels) and subtracting the background (a control channel without cells under the same conditions). We show below in Fig. S2 the raw data without background subtraction together without the background signal. The mean background of the image is equal to 132±2 (a.u.). A single cell image at the 20x magnification objective covers approx. 25px and has a maximum intensity of about 220±20, which means that about 2000 cells are needed in an image to reasonably emerge from the background. This cell number per image requires cell division (3 to 4 generations). Indeed, taking into account the total number of injected cells ($3x10^4$ cells per channel), no more than 150 cells can reach the surface in the area captured by one image (≈0.15 mm^2) at time t=0.

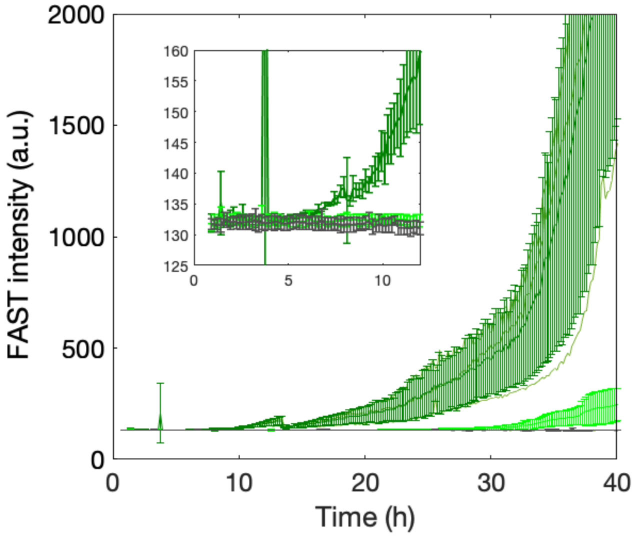

Figure S2: Background level : Fluorescence intensity of the background taken in the absence of cells (grey curves) is compared with sample signal, nonmotile cells injected at time t=0 (dark green) and motile cells (light green)without correction. Each averaged over 6 different positions from 2 channels. Error bars are standard deviations.

- **Motile and nonmotile cells exhibit similar growth rates**

In order to detect potential difference in the division rates of motile and nonmotile cells, we measured both strains optical densities over time in MB medium at 30°C using a microplate reader (Tecan TECAN Infinite M200 pro equipped with UV Xenon flashlamp light source). 10^6 cells (10^6cells/ml exponentially growing) were seeded in each well in triplicate and left to grow over night, taking one measurement every 10 min. The curves displayed in Fig. S3 show no significant difference between motile and nonmotile growth.

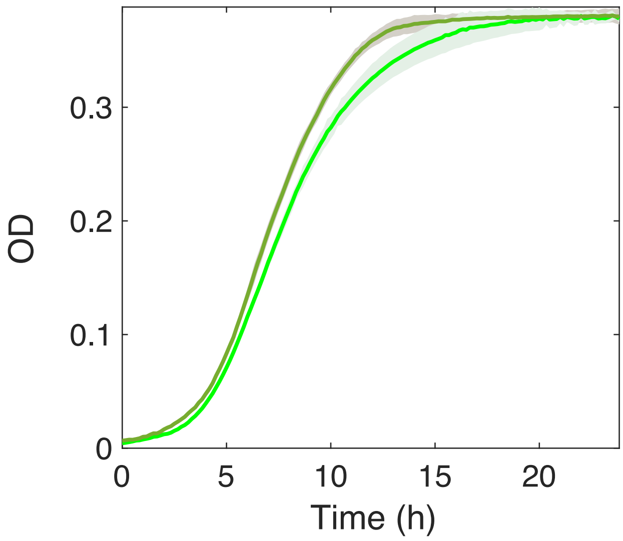

Figure S3: Motile and nonmotile cells have similar division rates under planktonic growth. Nonmotile cells (dark green) and motile cells (light green). 10^6 cells/ml were seeded in MB medium. Measurements performed in the triplicates in 48-wells plates. Curves represent the triplicate average shaded with the standard deviation of the data set.

- **Cell counts in the initial state**

The number of trajectories detected in the initial stage of biofilm formation when single cells can be delineated as explained in the text of our article is a proxy for the cell count of the surface. Fig. S4 shows its evolution over time for motile and nonmotile cells confirming the large discrepancy between the two.

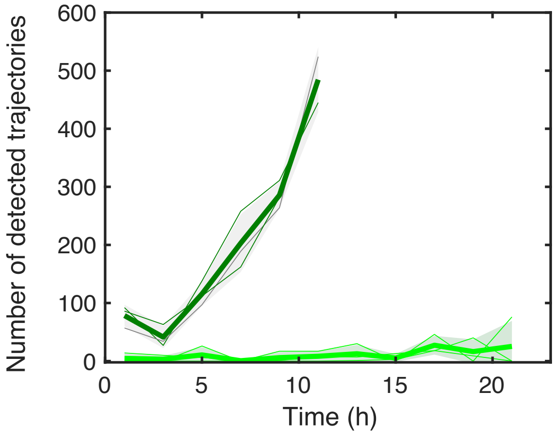

Figure S4: Cell counts on the surface : The number of cell trajectories detected over time for nonmotile (light green curve) and nonmotile (dark green curve) as explained in article ‘Materials and Methods’ section. Detection is performed on a 440x336 µm^2 image. Values are averaged over 3 positions. Error bars are standard deviations.

II. Biofilm development kinetic described as a logistic growth

In order to formalize the hypothesis of the inoculated population abundance on the biofilm development kinetic, we made the hypothesis that the biofilm under flow could be reasonably described using a logistic equation as follows :

$$S(t) = \frac{K}{1 + \left(\frac{K - S_0}{S_0}\right) \exp\left(-bt\right)}$$

The equation captures the exponential growth of the dividing population size, S(t) and the saturation imposed by environmental factors such as nutrient limitation, toxic metabolites buildup or steric constrains[5]. b is the growth rate, K, the environmental carrying capacity, i.e. the maximal size the population can be reached, and S_0, the size of the initial population. Fig. S5A shows a series of curves S(t) generated using same arbitrary values of K and b and different values of S_0, from 0.1 to 100, which illustrates how initial abundance decrease delays biofilm development.

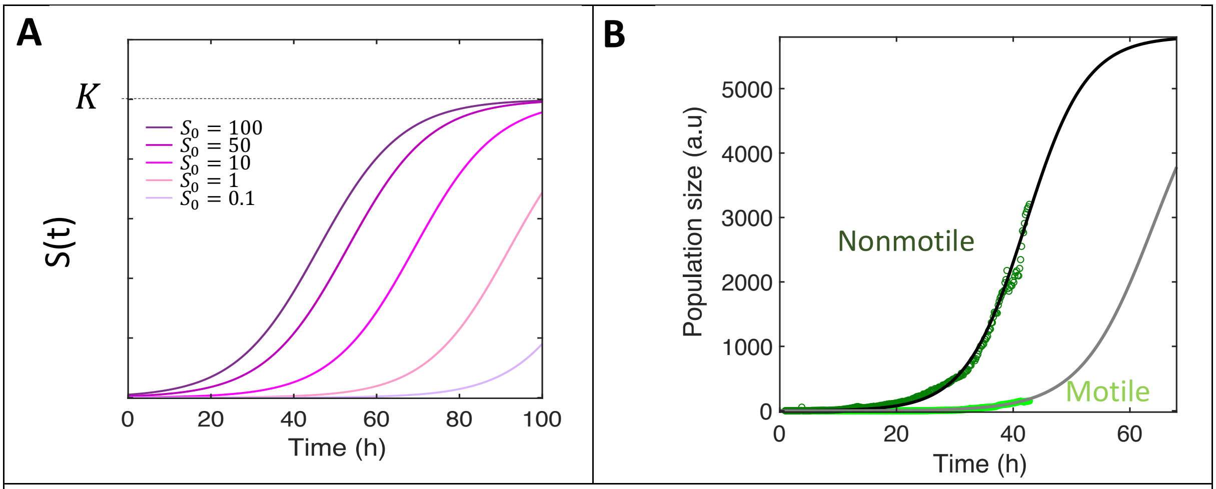

Figure S5: Biofilm development phenomenologically fits logistic growth hypothesis. (A) Typical logistic growth profiles within a three orders of magnitude range of initial population size S_0 (B) Adjustment of the experimental data with a logistic growth hypothesis[5] ; nonmotile cells biofilm data (dark green dot) are adjusted to a logistic profile with K= 5800 a.u.; b=0.19 h^{-1}(black line); motile cells data (light green dots) are adjusted using K=5800; b=0.17 h^{-1}.

To further investigate, the predictive quantitative power of our model, we tested the hypothesis of the logistic growth to account for our experimental data as a phenomenological guideline to score motile and nonmotile biofilms growth. Several parameter sets provided equally good quality adjustments with R-squared value >0.99. In particular, the parameter K accepted a whole range of values due the absence of description of the fully saturated level in the experiments. Nevertheless, assuming the same carrying capacity ($5.8x10^3$ in arbitrary units related to *E. coli* biofilm fluorescence signal) and similar growth rates (0.19 h^{-1} and 0.17 h^{-1} for nonmotile and motile biofilms, respectively, we obtained the adjustments displayed in Fig. S1B which provided an initial population sizes ratio (nonmotile to motile) of 12.

This ratio is approximately twice the ratio predicted by our sedimentation/diffusion model which suggests that the calculation slightly overestimates the number of cells that actually reach the surface by diffusion before flow starts. This might be due to the hypothesis of an adhesive first passage adhesion as explained above. Nevertheless, the whole picture strongly supports the hypothesis of development kinetic predominantly controlled by the initial abundance of the cells on the surface.

III. Settling model description

We aim to predict the bacteria settling kinetics to the bottom side of a chamber for a suspension of nonmotile bacteria. The chamber volume is V (height H and bottom surface S, V=SH). We assume:

- Adhesion to the bottom surface is immediate at first contact.
- No hydrodynamic effect when a cell approaches the bottom surface (no trapping, no bouncing).
- Initially, the spatial distribution of bacteria across the chamber is uniform.

For the sake of simplicity, the problem is restricted to 1D.

Stokes' law gives the terminal settling speed of a round particle in a viscous fluid:

$$V_s = \frac{2\,a^2 g \Delta\rho}{9\eta}$$

with a the particle radius, g is the gravitational acceleration, $\Delta\rho$ is the difference of density between particle and fluid, and η is the fluid viscosity. For a bacterial cell in water, a=1um, g=9.8m/s^2, $\Delta\rho$=80 kg/m^3, and η=10^{-3} Ns/m^2. This yields to V_s=0.18 µm/s.

At each time period dt, there are $n\delta V$ cells reaching the bottom surface, wit $n = N_0/(SH)$ the cell density, and $\delta V = V_S S\,dt$ the size of the micro-volume. This directly gives

$$N_s^g(t) = \frac{N_0}{H} V_S t$$

This is valid until the last cell reaches the surface (at time $t = H/V_S$). For $t > H/V_S$, $N_s^g(t)$ is constant and equal to N_0.

The Brownian motion of the nonmotile cells has been neglected. Indeed, expecting a diffusion coefficient of approx. 0.27 µm^2/s, a characteristic time to travel the 1mm of channel height, $t = \frac{l^2}{D}$ >1000h is obtained to be compared with the 56min of the settling at a velocity V_s equal to 18µm/s.

IV. Motile cell coefficient of diffusion

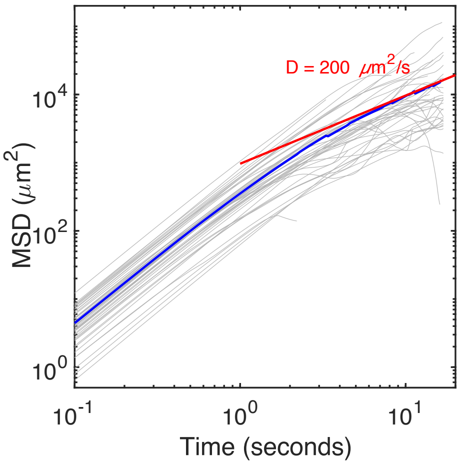

Figure S6: A small volume of exponentially growing E. coli cells were diluted in MB medium at 1.5x10^6 cells/ml and deposited in a small covered glass well for microscopy observations. Focus was made far enough from the surfaces and short high frequency (10 s^{-1}) sequences of high frequency images (10 s^{-1}) were recorded. 45 cells were tracked for 17 seconds. MSD (Mean squared displacement) are calculated on each trajectory (grey curves). All curves are averaged to obtain the mean MSD (blue curve). The late part of the mean MSD (between t=5s and t=17s) is fitted to a line of slope 1 to extract the diffusion coefficient D=200 μm^2/s.

V. Experimental geometric changes (H=250 μm and 1000 μm)

To test the settling hypothesis, we microfabricated smaller channels with 250 μm of height and examined the surface population over the first 90 min following the injection of the cells in the channel. We observed that the nonmotile cells followed a simple settling law both in the 1000 μm- and in the 250 μm-height channels. The height effect was also observed with the motile cells but the surface access kinetics were not reported by a random diffusion model taking sedimentation into account.

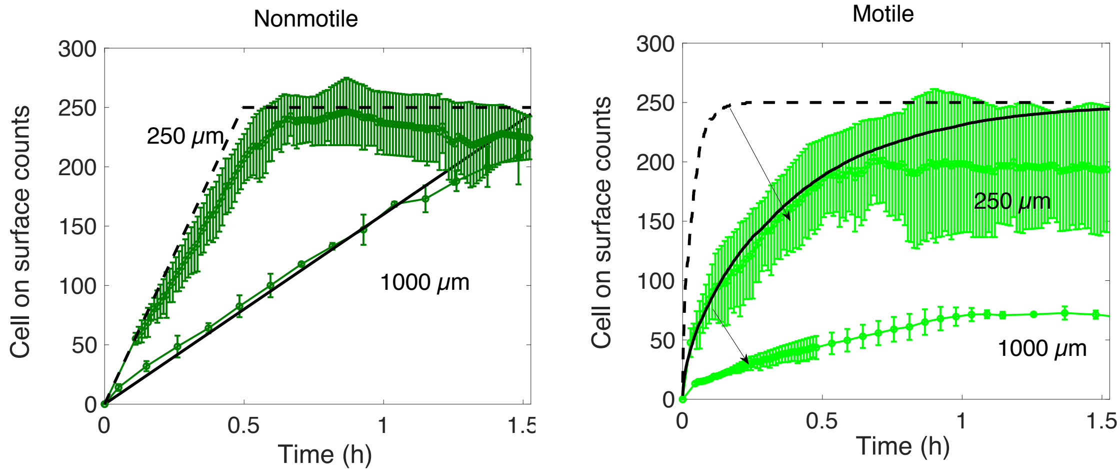

Figure S7: Channel height effect on settling and diffusion kinetics. Nonmotile (left panel) and Motile (right panel) cells are injected in channels of 250 µm or 1000µm height as indicated on the graphs at time t=0. Cell counts are performed over time on microscope images and plotted (green dots and lines) together with the calculated curves (in black lines lines).We used H=250µm (- -)or 1000µm (—); D=200 $\mu m^2/s$; total cell number=250 (expected from the cell suspension initial concentration equal to $1.6x10^6$ cells/ml and consistent with the nonmotile cell counts at the end of the incubation).

VI. Colonization of the pre-established four-species biofilm

We also examined the impact of an already established community on motile and nonmotile *E. coli* ability to colonize the surface. To this purpose, we initiated the 4-species biofilm formation in the channel at time t=0 and performed *E. coli* injection after 8, 20 and 36 hours which corresponded to 4-species community first climax, beginning of the second growth phase and established dynamical equilibrium, respectively. We observed that *E. coli* installation under these conditions was negligible in any of these conditions, never overpassing the level of the fluorescence background produced by the pre-settled community. In each case, we measured FAST fluorescence 40 hours after *E. coli* injection in the pre-colonized channel and observed no difference between motile and nonmotile cell samples. We concluded that the initial surface coverage with a biofilm prevented *E. coli* installation regardless its motility.

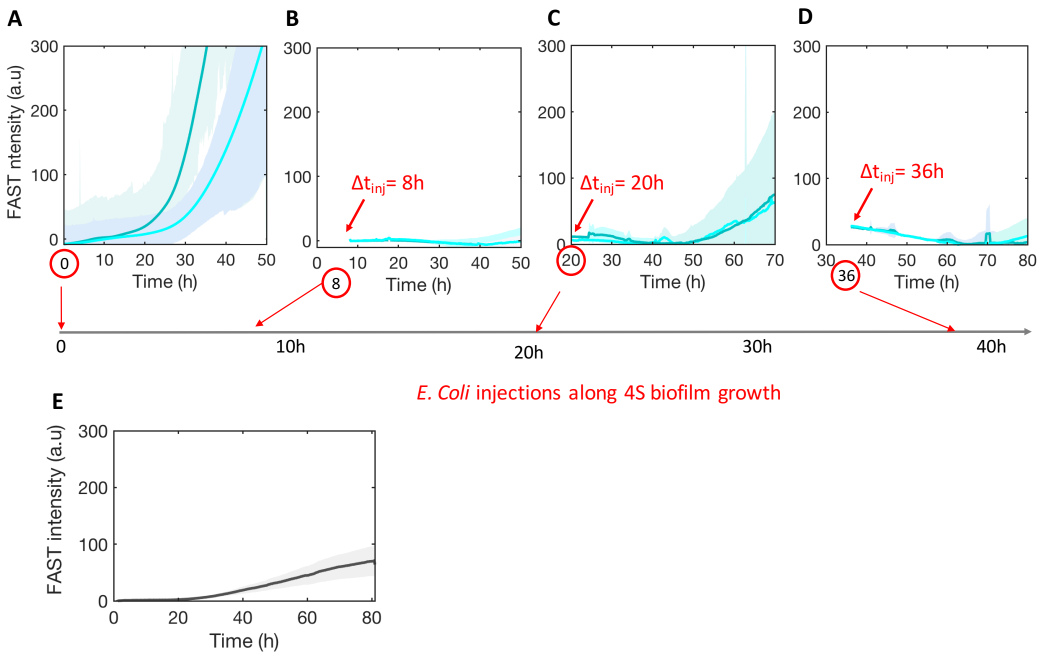

Figure S8: ***E. coli* fails to colonize surface with a pre-establish multispecies biofilm.** FAST fluorescence intensity on the channel surface — which report E. coli biofilm formation — is shown after the injection of *E. coli* in the channel where a 4S community has established for 0h (A), 8h (B), 20h (C) and 36h (D) for motile (light cyan) and nonmotile (cyan) cells. The time scale refers to 4S development with t=0 corresponding to 4S biofilm initiation. *E. coli* injections are mentioned with red arrows on the graphs. The 4S community FAST background (E) coming from its autofluorescence has been subtracted from all the signals. Only the co-colonizing situation corresponding to injection at time t=0 displays significant *E. coli* colonization. Curves are from the average intensity of 2 distinct positions in 3 different channels (3 biological replicates) with the shaded area representing the standard deviation.

Supplementary data references

1 Ghigo, J. M. Natural conjugative plasmids induce bacterial biofilm development. *Nature* **412**, 442-445 (2001).

2 Plamont, M. A. *et al.* Small fluorescence-activating and absorption-shifting tag for tunable protein imaging in vivo. *Proc Natl Acad Sci U S A* **113**, 497-502, doi:1513094113 (2016).

3 Lagendijk, E. L., Validov, S., Lamers, G. E., de Weert, S. & Bloemberg, G. V. Genetic tools for tagging Gram-negative bacteria with mCherry for visualization in vitro and in natural habitats, biofilm and pathogenicity studies. *FEMS Microbiol Lett* **305**, 81-90, doi:10.1111/j.1574-6968.2010.01916.x (2010).

4 Barker, C. S., Pruss, B. M. & Matsumura, P. Increased motility of Escherichia coli by insertion sequence element integration into the regulatory region of the flhD operon. *J Bacteriol* **186**, 7529-7537, doi:10.1128/JB.186.22.7529-7537.2004 (2004).

5 Allen, R. J. & Waclaw, B. Bacterial growth: a statistical physicist's guide. *Rep Prog Phys* **82**, 016601, doi:10.1088/1361-6633/aae546 (2019).
